# Comparative Analysis of Clinical Characteristics in Multi-organ and Single-organ Involvement of IgG4-Related Disease: A Single-center Retrospective Study

**DOI:** 10.34172/aim.34027

**Published:** 2025-05-01

**Authors:** Dong-Ge Han, Chun-Lin Ying, Zi-Ping Cai, Qiao-Yun Tong, Wei Liu

**Affiliations:** ^1^The First College of Clinical Medical Science, China Three Gorges University, Yichang, China; ^2^Institute of Digestive Disease, China Three Gorges University, Yichang, China; ^3^Department of Gastroenterology, Yichang Central People’s Hospital, Yichang, China

**Keywords:** Clinical characteristics, Immunoglobulin G4-related disease, Organ involvement

## Abstract

**Background:**

Immunoglobulin G4-related disease (IgG4-RD) is a rare, chronic inflammatory condition characterized by fibrosis and tendency for multi-organ involvement. This study aims to analyze the clinical characteristics associated with multi-organ versus single-organ involvement in IgG4-RD, thereby enhancing clinicians’ understanding of the differences between these two patient groups and ultimately improving patient prognosis.

**Methods:**

We performed a retrospective analysis of clinical data from 82 patients diagnosed with IgG4-RD admitted to Yichang Central People’s Hospital between January 2019 and December 2024.

**Results:**

Among the 82 patients diagnosed with IgG4-RD, 47 patients (57.32%) exhibited involvement of multiple organs. The incidence of multi-organ involvement was significantly higher in male patients than female patients [63.49% vs. 36.84%, odds ratio (OR): 2.98, 95% confidence intervals (CI): 1.03–8.64, *P*<0.05]. The misdiagnosis rate in the multi-organ involvement group was significantly higher than that in the single-organ involvement group (29.79% vs. 8.57%, OR: 4.525, 95% CI: 1.19–17.26, *P*<0.05). In patients with involvement of the pancreas (72.50% vs. 42.86%, OR: 3.515, 95% CI: 1.39–8.86, *P*<0.05), or lymph nodes (83.72% vs. 28.21%, OR: 13.091, 95% CI: 4.50–38.11, *P*<0.05), the incidence of additional organ involvement was significantly higher than those with involvement of other organs. The eosinophil percentage [median difference (Hodges-Lehmann): 1.60%, 95% CI: 0.40–2.80, *P*<0.05], absolute eosinophil count [median difference (Hodges-Lehmann): 0.10×10^9^/L, 95% CI: 0.30–0.16, *P*<0.05], serum immunoglobulin G (IgG) levels [median difference (Hodges-Lehmann): 4.10 g/L, 95% CI: 0.10–7.80, *P*<0.05], and erythrocyte sedimentation rate (ESR) [median difference (Hodges-Lehmann): 30.50 mm/h, 95% CI: 13.00–48.00, *P*<0.05] were significantly higher in the multi-organ involvement group compared to the single-organ involvement group. There was a positive correlation between the number of involved organs and ESR (*r*=0.404, 95% CI: 0.166–0.597, *P*=0.001), eosinophil percentage (*r*=0.287, 95% CI: 0.068–0.480, *P*=0.009), absolute eosinophil count (*r*=0.293, 95% CI: 0.075–0.485, *P*=0.007), serum IgG levels (*r*=0.370, 95% CI: 0.130–0.570, *P*=0.003), and serum IgG4 levels (*r*=0.370, 95% CI: 0.130–0.570, *P*=0.003).

**Conclusion:**

The clinical features associated with multi-organ involvement in IgG4-RD are characterized by significant diversity and complexity. Clinicians must enhance their understanding of the characteristics associated with multi-organ involvement to more effectively improve patient prognosis.

## Introduction

 Immunoglobulin G4-related disease (IgG4-RD) is an immune-mediated chronic inflammatory and fibrotic condition that has garnered significant attention in recent years.^1-4^ Epidemiological studies on IgG4-RD have predominantly centered on the Japanese population, whereas there remains a significant paucity of data from China. According to a nationwide survey conducted in Japan in 2009, the annual incidence of IgG4-RD was reported to range from 0.28 to 1.08 cases per 100 000 individuals, with an estimated prevalence of 6.2 per 100 000 population.^5^ IgG4-RD is a systemic condition that can involve nearly all organ systems, with the pancreas, bile duct, salivary glands, lacrimal glands, and lymph nodes being the primary sites of involvement.^6-9^ Furthermore, other organs involved in IgG4-RD include the lung, liver, gastrointestinal tract, pituitary gland, dura mater, kidney and so on.^10-14^ A minority of patients exhibit involvement of a single organ, whereas the majority experience concurrent or sequential involvement of multiple organs. The clinical features of multi-organ involvement in IgG4-RD are more complex than those associated with single-organ involvement, exhibiting a diverse array of manifestations and interwoven clinical characteristics.^15,16^ This complexity underscores the imperative for a more thorough and comprehensive understanding of the clinical characteristics associated with both multi-organ and single-organ involvement in IgG4-RD. Although previous studies have described the organ involvement patterns in IgG4-RD,^17-19^ systematic comparisons of the clinical features between multi-organ and single-organ involvement remain relatively scarce. The current literature focuses primarily on the individual manifestations of various organs,^16,20,21^ lacking a comprehensive analysis of the potential differences between the two patterns of involvement. Therefore, in this study, we retrospectively analyzed the clinical data of 82 patients diagnosed with IgG4-RD in a comprehensive tertiary hospital (Yichang Central People’s Hospital) in China from January 2019 to December 2024. This study aims to conduct a comprehensive analysis of the clinical characteristics of patients with multi-organ involvement compared to those with single-organ involvement in IgG4-RD. By enhancing clinicians’ understanding of the differences between these two patient groups, we seek to provide a robust scientific foundation and practical clinical guidance, ultimately enhancing patient prognosis.

## Patients and Methods

###  Patients 

 This investigation included 82 patients diagnosed with IgG4-RD at Yichang Central People’s Hospital (hereafter referred to as “our institution”) between January 2019 and December 2024. The study was conducted in accordance with the ethical principles outlined in the Declaration of Helsinki and received an exemption from the Medical Ethics Committee of our institution due to its retrospective nature (approval number: 2024-361-01). Considering the retrospective design of the study and the use of anonymized patient data, the ethics committee determined that informed consent from participants was not required.

###  Study Design

 This study adhered to the STROBE guidelines for reporting observational research.

###  Inclusion Criteria

 The diagnostic criteria were as follows: (1) The 2020 revised comprehensive diagnostic (RCD) criteria for IgG4-RD^22^; 2. The 2019 American College of Rheumatology (ACR)/European League Against Rheumatism (EULAR) Classification Criteria for IgG4-RD.^23^ The diagnosis can be confirmed if one of the above diagnostic criteria is met. In this study, all patients underwent a comprehensive evaluation by two rheumatology specialists, one relevant subspecialist and one radiology expert to determine the number of involved organs.

###  Exclusion Criteria

 Patients with a confirmed diagnosis of a tumor or imaging findings suggestive of a tumor that had not been definitively excluded; patients with confirmed diagnoses of Castleman’s disease, primary sclerosing cholangitis, Wegener’s granulomatosis, tuberculosis, or other similar conditions; and patients with incomplete medical records lacking essential clinical data were excluded from this study.

###  Laboratory Tests and Imaging

 Upon admission, all patients were tested for a complete blood count. Additionally, serum tests were conducted as follows: immunoglobulin G4 (IgG4) was measured in 67 patients; immunoglobulin G (IgG) was measured in 64 patients, complement C3 (C3), complement C4 (C4), and erythrocyte sedimentation rate (ESR) were assessed in 63 patients; and C-reactive protein (CRP) was evaluated in 77 patients.

 All patients underwent imaging examinations, which included computed tomography (CT), magnetic resonance imaging (MRI) or ultrasonography. Additionally, a subset of patients underwent further evaluations with magnetic resonance cholangiopancreatography (MRCP) or 18F fluorodeoxyglucose positron emission tomography/computed tomography (18F-FDG PET/CT).

###  Definitions

 The normal reference range for serum IgG4 in our institution is defined as 0.03‒2.01 g/L, with 2.01 g/L serving as the upper limit of normal (ULN) for serum IgG4. In our study, involvement of only one organ was defined as single-organ involvement, whereas involvement of two or more organs was defined as multi-organ involvement.

###  Statistics

 The Shapiro-Wilk test was employed to assess the normality of continuous variables. Normally distributed continuous variables were presented as mean ± standard deviation (SD), whereas non-normally distributed variables were expressed as median with interquartile range (IQR). Categorical variables were summarized using frequency distributions and percentages. For comparisons of continuous variables, the *t*-test was applied for normally distributed data, while the Mann-Whitney U test was used for non-normally distributed data. For the Mann-Whitney U test, we employed the Hodges-Lehmann method to estimate the confidence intervals (CIs). Levene’s test was conducted to assess the homogeneity of variances prior to the *t*-test; if this assumption was violated, Welch’s *t*-test was used instead. The chi-square test was employed for categorical variables with sufficient expected cell counts, while Fisher’s exact test was used for those with insufficient expected cell counts. During the chi-squared analysis, we calculated the odds ratios (OR) and their corresponding CI. To manage multiple comparisons, a Bonferroni correction was applied as needed to adjust the significance threshold, thereby controlling the family-wise error rate and maintaining the overall Type I error probability below 0.05. Correlation analyses were performed using Pearson’s correlation coefficient for continuous variables following a normal distribution and Spearman’s rank correlation coefficient for variables with non-normal distributions or ordinal scales. The independence of observations was confirmed to ensure analytical validity. Rigor was applied during data collection to ensure the independence of observations, with careful selection of subjects to prevent mutual influence. The handling of missing data was meticulously assessed. For data missing completely at random (MCAR), listwise deletion was implemented, while multiple imputation techniques were employed for data missing at random (MAR). Statistical analyses were conducted using SPSS Statistics version 27.0 (IBM Corp., Armonk, NY, USA). All figures were generated with GraphPad Prism version 10.4 (GraphPad Software Inc., La Jolla, CA, USA). All analyses were two-tailed and performed with a 95% CI, with *P*-values less than 0.05 regarded as statistically significant.

###  Power Calculation

 This study employed the PASS software (version 2021, NCSS, LLC, Kaysville, UT, USA) to conduct a post hoc power analysis, aimed at assessing the statistical power of the research. At a significance level (α) of 0.05 and an effect size (Cohen’s d, Proportion Difference, Hodges-Lehmann estimator or Spearman’s r) estimated from preliminary data, the statistical power was analyzed under the sample sizes of 47 cases in the multi-organ involvement group and 35 cases in the single-organ involvement group. In the correlation analysis, the analyses for serum IgG levels (89.76%) and ESR (94.38%) exhibited statistical power exceeding 80.00%, while the results for absolute eosinophil count (73.94%), eosinophil percentage (73.58%), and serum IgG4 levels (74.34%) demonstrated statistical power approaching 80.00%. In the analysis using the Mann-Whitney U test for two independent samples, the results for ESR (96.08%) and serum IgG levels (91.33%) demonstrated statistical power exceeding 80.00%. In contrast, the analyses for eosinophil percentage (73.30%) and absolute eosinophil count (71.36%) showed statistical power approaching 80.00%. In the Chi-square analysis, the results for pancreatic involvement (81.33%) and lymph node involvement (89.39%) demonstrated statistical power exceeding 80.00%, while the analysis for gender (70.65%) and misdiagnosis rate (73.71%) showed statistical power approaching 80.00%. It is important to acknowledge that while the statistical power for certain variables was below 80.00%, IgG4-RD is a rare condition, with an incidence rate ranging from 0.28 to 1.08 cases per 100 000 person-years and a prevalence of 0.062%.^5^ Consequently, this study maintains adequate statistical power to identify correlations among the target variables and differences between groups.

## Results

###  Baseline Characteristics

 A total of 82 adult patients diagnosed with IgG4-RD were included in this study, with a mean age at diagnosis of 59.13 ± 10.75 years (range: 35–89 years). Among these patients, 63 (76.83%) were male and 19 (23.17%) were female, yielding a male-to-female ratio of 3.32:1. On average, each patient had two involved organs (IQR: 1–3). Among the 35 (42.68%) patients diagnosed with IgG4-RD who exhibited involvement of a single organ, the mean age at diagnosis was 58.66 ± 11.08 years. This cohort consisted of 23 (65.71%) males and 12 (34.29%) females. Among the 47 (57.32%) patients with multi-organ involvement, the mean age at diagnosis was 59.49 ± 10.61 years. The cohort comprised 40 (85.11%) males and 7 (14.89%) females (Table 1). The incidence of multi-organ involvement was significantly higher in male patients than female patients [63.49% (40/63) vs. 36.84% (7/19), OR: 2.98, 95% CI: 1.03–8.64,* P*< 0.05].

**Table 1:** Comparison of Baseline Characteristics between Patients with Single-organ and Multi-organ Involvement in IgG4-RD

**Items**	**Total** **(n=82)**	**Single-organ Involvement** **(n=35)**	**Multi-organ Involvement** **(n=47)**	***P*** ** Value**
Age, mean ± SD, years	59.13 ± 10.75	58.66 ± 11.08	59.49 ± 10.61	0.731
Sex, n (%)				
Male	63 (76.83%)	23 (65.71%)	40 (85.11%)	0.040*
Female	19 (23.17%)	12 (34.29%)	7 (14.89%)	
Misdiagnosis, n (%)	17 (20.73%)	3 (8.57%)	14 (29.79%)	0.019*
Smoking history, n (%)	34 (41.46%)	14 (40.00%)	20 (42.55%)	0.816
Drinking history, n (%)	22 (26.83%)	7 (20.00%)	15 (31.91%)	0.228
Allergy history, n (%)	6 (7.32%)	2 (5.71%)	4 (8.51%)	0.958
Hypertension history, n (%)	14 (17.07%)	5 (14.29%)	9 (19.15%)	0.563
Diabetes history, n (%)	8 (9.76%)	4 (11.43%)	4 (8.51%)	0.949

Data are n (%) unless otherwise indicated. *Values indicate statistical significance.

 Among 82 patients with IgG4-RD, 17 (20.73%) patients were initially misdiagnosed as other conditions, with tumors being the most common misdiagnosis (14 patients, 82.35%). The misdiagnosis rate in the multi-organ involvement group was significantly higher than that in the single-organ involvement group (29.79% vs. 8.57%, OR: 4.525, 95% CI: 1.19–17.26, *P*< 0.05) (Table 1).

 In this study, the most common initial symptom among the 82 patients with IgG4-RD was abdominal pain (31 patients, 38.80%). This was followed by jaundice (18 patients, 21.95%), abdominal distension (11 patients, 13.41%), facial mass (10 patients, 12.20%), fatigue (6 patients, 7.32%), loss of appetite (6 patients, 7.32%), cough (6 patients, 7.32%), and lymph node enlargement (5 patients, 6.10%). Additionally, 9 patients (10.98%) were asymptomatic. Very few patients experienced expectoration, hemoptysis, bilateral lower limb edema, eyelid swelling, diarrhea, rash and dysuria. It is noteworthy that patients with IgG4-RD may present with a multitude of concurrent initial symptoms.

###  Involved Organs

 In this study, the three most commonly involved organs were the lymph nodes (43 patients, 52.44%), pancreas (40 patients, 48.78%), and bile ducts (21 patients, 25.61%). Following these, the disease also involved the lungs (17 patients, 20.73%), kidneys (9 patients, 10.98%), salivary glands (10 patients, 12.20%), retroperitoneal fibrosis (6 patients, 7.32%), liver (4 patients, 4.88%), gastrointestinal tract (3 patients, 3.66%), lacrimal glands (4 patients, 4.88%), pleura (1 patient, 1.22%), mesentery (1 patient, 1.22%), skin (1 patient, 1.22%), breast (1 patient, 1.22%) and paranasal sinus (1 patient, 1.22%). In patients with involvement of the pancreas (72.50% vs. 42.86%, OR: 3.515, 95% CI: 1.39–8.86,* P* < 0.05), or lymph nodes (83.72% vs. 28.21%, OR: 13.091, 95% CI: 4.50–38.11,* P* < 0.05), the incidence of additional organ involvement was significantly higher than those with involvement of other organs (Table 2). Although this study observed that patients with bile duct and kidney involvement were all categorized as having multi-organ involvement, and statistical analyses indicated significance, we cautiously acknowledge that the relatively small sample size and the limited number of cases involving bile duct and kidney involvement may introduce the possibility of false-positive results.

**Table 2:** Incidence of Additional Organ Involvement among Patients with Various Types of Involved Organs

**Items**	**Total**	**Single-organ Involvement**^a^	**Multi-organ Involvement**^a^	***P*** ** Value**
Pancreas	40 (48.78%)	11 (27.50%)	29 (72.50%)	0.007*
Bile duct	21 (25.61%)	0 (0.00%)	21 (100.00%)	< 0.001
Liver	4 (4.88%)	1 (25.00%)	3 (75.00%)	0.830
Gastrointestinal tract	3 (3.66%)	1 (33.33%)	2 (66.67%)	1.000
Lung	17 (20.73%)	5 (29.41%)	12 (70.59%)	0.214
Pleura	1 (1.22%)	0 (0.00%)	1 (100.00%)	1.000
Salivary gland	10 (12.20%)	5 (50.00%)	5 (50.00%)	0.874
Lacrimal gland	4 (4.88%)	2 (50.00%)	2 (50.00%)	1.000
Kidney	9 (10.98%)	0 (0.00%)	9 (100.00%)	0.017
Retroperitoneal fibrosis	6 (7.32%)	2 (33.33%)	4 (66.67%)	0.958
Mesentery	1 (1.22%)	0 (0.00%)	1 (100.00%)	1.000
Lymph node	43 (52.44%)	7 (16.28%)	36 (83.72%)	< 0.001*
Skin	1 (1.22%)	0 (0.00%)	1 (100.00%)	1.000
Breast	1 (1.22%)	0 (0.00%)	1 (100.00%)	1.000
Paranasal sinus	1 (1.22%)	1 (100.00%)	0 (0.00%)	0.882

Data are n (%) unless otherwise indicated. * Values indicate statistical significance. ^a^ Involvement of additional organs in patients is indicative of multi-organ involvement, whereas involvement limited to a single organ is considered single-organ involvement.

###  Comparative Analysis of Laboratory Findings in Patients with Single-organ Versus Multi-organ involvement

**Figure 1:**
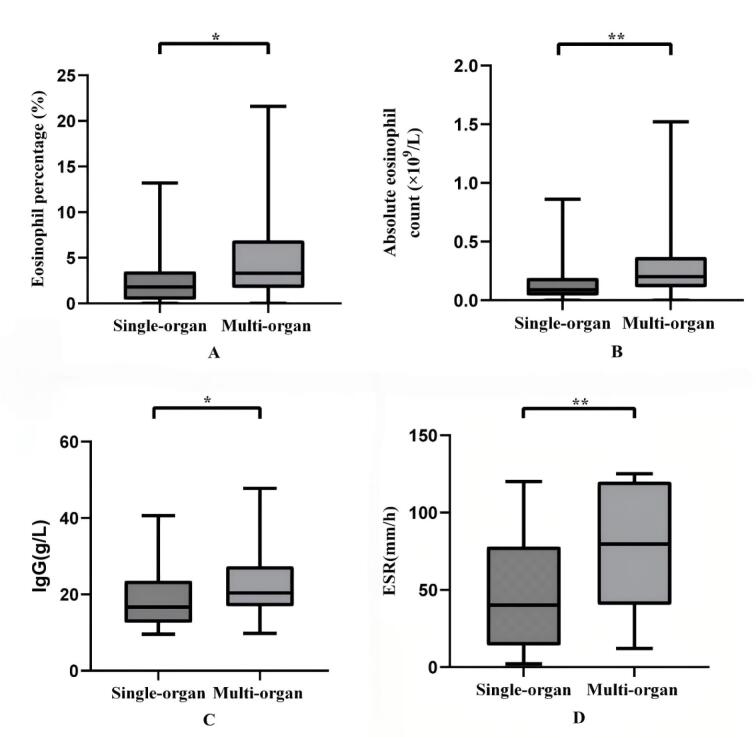
Levels of Eosinophil Percentage **(A)**, Absolute Eosinophil Count **(B)**, Immunoglobulin G (IgG), **(C) **and Erythrocyte Sedimentation Rate (ESR), **(D) **in Peripheral Blood with Single-organ Involvement and Multi-organ Involvement. The eosinophil percentage, absolute eosinophil count, serum IgG levels and ESR were significantly higher in the multi-organ involvement group compared to the single-organ involvement group (*P*< 0.05)

 Laboratory findings in the multi-organ involvement group compared to the single-organ involvement group are presented in Table 3. In this study, 82 patients with IgG4-RD underwent evaluation of peripheral blood eosinophil percentages and absolute eosinophil counts. The median eosinophil percentage was 2.75% (IQR: 1.15%–5.38%) and the median absolute eosinophil count was 0.15 (IQR: 0.05–0.31) × 10^9^/L. Sixty-four patients with IgG4-RD underwent evaluation of IgG and 63 patients underwent evaluation of ESR. The median IgG was 18.75 (IQR: 14.83–27.05) g/L and the median ESR was 64.00 (IQR: 32.00–100.00) mm/h. We observed that the median eosinophil percentage [3.30 (IQR: 1.70–6.90) vs. 1.80 (IQR: 0.40–3.50)%, median difference (Hodges-Lehmann): 1.60%, 95% CI: 0.40–2.80, *P* < 0.05] (Figure 1A), absolute eosinophil count [0.20 (IQR: 0.11–0.37) vs. 0.09 (IQR: 0.04–0.19) × 10^9^/L, median difference (Hodges-Lehmann): 0.10 × 10^9^/L, 95% CI: 0.30–0.16, *P* < 0.05] (Figure 1B), IgG [20.40 (IQR: 16.85–27.40) vs. 16.69 (IQR: 12.60–23.58) g/L, median difference (Hodges-Lehmann): 4.10 g/L, 95% CI: 0.10–7.80,* P* < 0.05] (Figure 1C), and ESR [79.50 (IQR: 40.25–120.00) vs. 40.00 (IQR: 14.00–78.00) mm/h, median difference (Hodges-Lehmann): 30.50 mm/h, 95% CI: 13.00–48.00,* P* < 0.05] (Figure 1D) were significantly higher in the multi-organ involvement group compared to the single-organ involvement group.

 Serum IgG4 levels were measured in 67 out of 82 patients diagnosed with IgG4-RD. In this study, serum IgG4 levels were categorized into four groups based on the ULN: IgG4 ≤ ULN (11 patients, 16.42%), ULN < IgG4 < 2 × ULN (18 patients, 26.87%), 2 × ULN ≤ IgG4 < 5 × ULN (24 patients, 35.82%), and IgG4 ≥ 5 × ULN (14 patients, 20.90%). The majority of serum IgG4 levels were observed in 2 × ULN ≤ IgG4 < 5 × ULN.

**Table 3:** Comparison of Laboratory Findings between Patients with Single-organ and Multi-organ Involvement

**Items**	**Single-organ Involvement** **(** ***n*** **=35)**	**Multi-organ Involvement** **(** ***n*** **=47)**	***P *** **Value**
Red blood cells ( × 10^12^/L)	4.20 (3.79 – 4.51)	3.96 (3.59 – 4.39)	0.391
Platelets ( × 10^9^/L)	195.00 (135.00 – 248.00)	208.00 (151.00 – 281.00)	0.536
Hemoglobin (g/L)	122.63 ± 26.88	118.94 ± 18.70	0.465
White blood cells ( × 10^9^/L)	5.76 (4.27 – 8.37)	6.33 (4.61 – 7.47)	0.959
Neutrophil percentage (%)	66.96 ± 13.09	62.30 ± 12.33	0.103
Absolute neutrophil count ( × 10^9^/L)	3.82 (2.63 – 6.76)	3.52 (2.89 – 4.91)	0.574
Basophil percentage (%)	0.40 (0.10 – 0.60)	0.50 (0.20 – 0.80)	0.200
Absolute basophil count ( × 10^9^/L)	0.02 (0.01 – 0.03)	0.03 (0.02 – 0.04)	0.098
Eosinophil percentage (%)	1.80 (0.40 – 3.50)	3.30 (1.70 – 6.90)	0.010*
Absolute eosinophil count ( × 10^9^/L)	0.09 (0.04 – 0.19)	0.20 (0.11 – 0.37)	0.006*
Lymphocyte percentage (%)	21.42 ± 8.96	22.97 ± 7.63	0.401
Absolute lymphocyte count ( × 10^9^/L)	1.17 (0.96 – 1.46)	1.28 (1.02 – 1.73)	0.259
Monocyte percentage (%)	8.20 (6.50 – 10.10)	8.20 (7.20 – 10.30)	0.442
Absolute monocyte count ( × 10^9^/L)	0.47 (0.30 – 0.63)	0.53 (0.43 – 0.68)	0.356
Serum IgG4 levels (*n*, %)^a^	**n=30**	**n=37**	
IgG4 ≤ ULN	6 (20.00%)	5 (13.51%)	0.068
ULN < IgG4 < 2 × ULN	9 (30.00%)	9 (24.32%)	
2 × ULN ≤ IgG4 < 5 × ULN	13 (43.33%)	11 (29.73%)	
IgG4 ≥ 5 × ULN	2 (6.67%)	12 (32.43%)	
IgG (g/L)^b^	16.69 (12.60 – 23.58)	20.40 (16.85 – 27.40)	0.046*
C3 (g/L)^c^	0.98 ± 0.31	1.02 ± 0.37	0.640
C4 (g/L)^d^	0.22 ± 0.12	0.22 ± 0.13	0.796
ESR (mm/h)^e^	40.00 (14.00–78.00)	79.50 (40.25–120.00)	0.002*
CRP (mg/L)^f^	8.11 (2.19–37.32)	7.06 (3.22–22.96)	0.804

ULN, Upper limit of normal; IgG4, Immunoglobulin G4; IgG, Immunoglobulin G; C3, Complement C3; C4, Complement C4; ESR, Erythrocyte sedimentation rate; CRP, C-reactive protein. Date are *n* (%), mean ± SD, or median (IQR), unless otherwise indicated. * Values indicate statistical significance. ^a^A total of 67 patients underwent serum IgG4 testing. ^b^A total of 64 patients underwent serum IgG testing. ^c^A total of 63 patients underwent C3 testing. ^d^A total of 63 patients underwent C4 testing. ^e^A total of 63 patients underwent ESR testing. ^f^A total of 77 patients underwent CRP testing.

 In the single-organ involvement group, 30 patients underwent serum IgG4 testing, with the majority of serum IgG4 levels falling within the range of 2 × ULN ≤ IgG4 < 5 × ULN (13 patients, 43.33%). In the multi-organ involvement group, serum IgG4 testing was conducted for 37 patients, and a significant majority exhibited IgG4 ≥ 5 × ULN (12 patients, 32.43%) (Table 3). The incidence of multi-organ involvement [85.71% (12/14) vs. 45.45% (5/11) vs. 50.00% (9/18) vs. 45.83% (11/24)] in IgG4 ≥ 5 × ULN was significantly higher compared to the other three groups. However, this study did not reveal a significant statistical difference in serum IgG4 levels between the multi-organ involvement group and the single-organ involvement group (*P*> 0.05).

###  Correlation Analysis between the Number of Involved Organs and Laboratory Findings

 We performed a Spearman correlation analysis to evaluate the relationship between the number of involved organs and laboratory findings in 82 patients diagnosed with IgG4-RD (Table 4). Notably, the number of involved organs demonstrated a moderate positive correlation with ESR (*r*= 0.404, 95% CI: 0.166–0.597, *P*= 0.001). However, the number of involved organs demonstrated a weak positive correlation with the eosinophil percentage (*r* = 0.287, 95% CI: 0.068–0.480, *P*= 0.009), absolute eosinophil count (*r*= 0.293, 95% CI: 0.075–0.485, *P*= 0.007), serum IgG4 levels (*r*= 0.296, 95% CI: 0.053–0.506, *P*= 0.015), and serum IgG levels (*r*= 0.370, 95% CI: 0.130–0.570, *P*= 0.003).

**Table 4:** Correlation Analysis between the Number of Involved Organs and Laboratory Findings

**Items**	***r *** **Value**	***P *** **Value**
Red blood cells ( × 10^12^/L)	-0.084	0.451
Platelets ( × 10^9^/L)	0.011	0.925
Hemoglobin (g/L)	-0.141	0.208
White blood cells ( × 10^9^/L)	-0.007	0.950
Neutrophil percentage (%)	-0.160	0.151
Absolute neutrophil count ( × 10^9^/L)	-0.078	0.487
Basophil percentage (%)	0.135	0.227
Absolute basophil count ( × 10^9^/L)	0.180	0.106
Eosinophil percentage (%)	0.287	0.009*
Absolute eosinophil count ( × 10^9^/L)	0.293	0.007*
Lymphocyte percentage (%)	0.068	0.546
Absolute lymphocyte count ( × 10^9^/L)	0.108	0.334
Monocyte percentage (%)	0.096	0.389
Absolute monocyte count ( × 10^9^/L)	0.084	0.454
Serum IgG4 levels (n, %)^a^		
IgG4 ≤ ULN	0.296	0.015*
ULN < IgG4 < 2 × ULN		
2 × ULN ≤ IgG4 < 5 × ULN		
IgG4 ≥ 5 × ULN		
IgG (g/L)^b^	0.370	0.003*
C3 (g/L)^c^	-0.098	0.445
C4 (g/L)^d^	-0.190	0.135
ESR (mm/h)^e^	0.404	0.001*
CRP (mg/L)^f^	0.002	0.984

ULN, Upper limit of normal; IgG4, Immunoglobulin G4; IgG, Immunoglobulin G; C3, Complement C3; C4, Complement C4; ESR, Erythrocyte sedimentation rate; CRP, C-reactive protein. *Values indicate statistical significance. ^a^A total of 67 patients underwent serum IgG4 testing. ^b^A total of 64 patients underwent serum IgG testing. ^c^A total of 63 patients underwent C3 testing. ^d^A total of 63 patients underwent C4 testing. ^e^A total of 63 patients underwent ESR testing. ^f^A total of 77 patients underwent CRP testing.

###  Features of Imaging

####  Pancreas and Bile Duct

**Figure 2:**
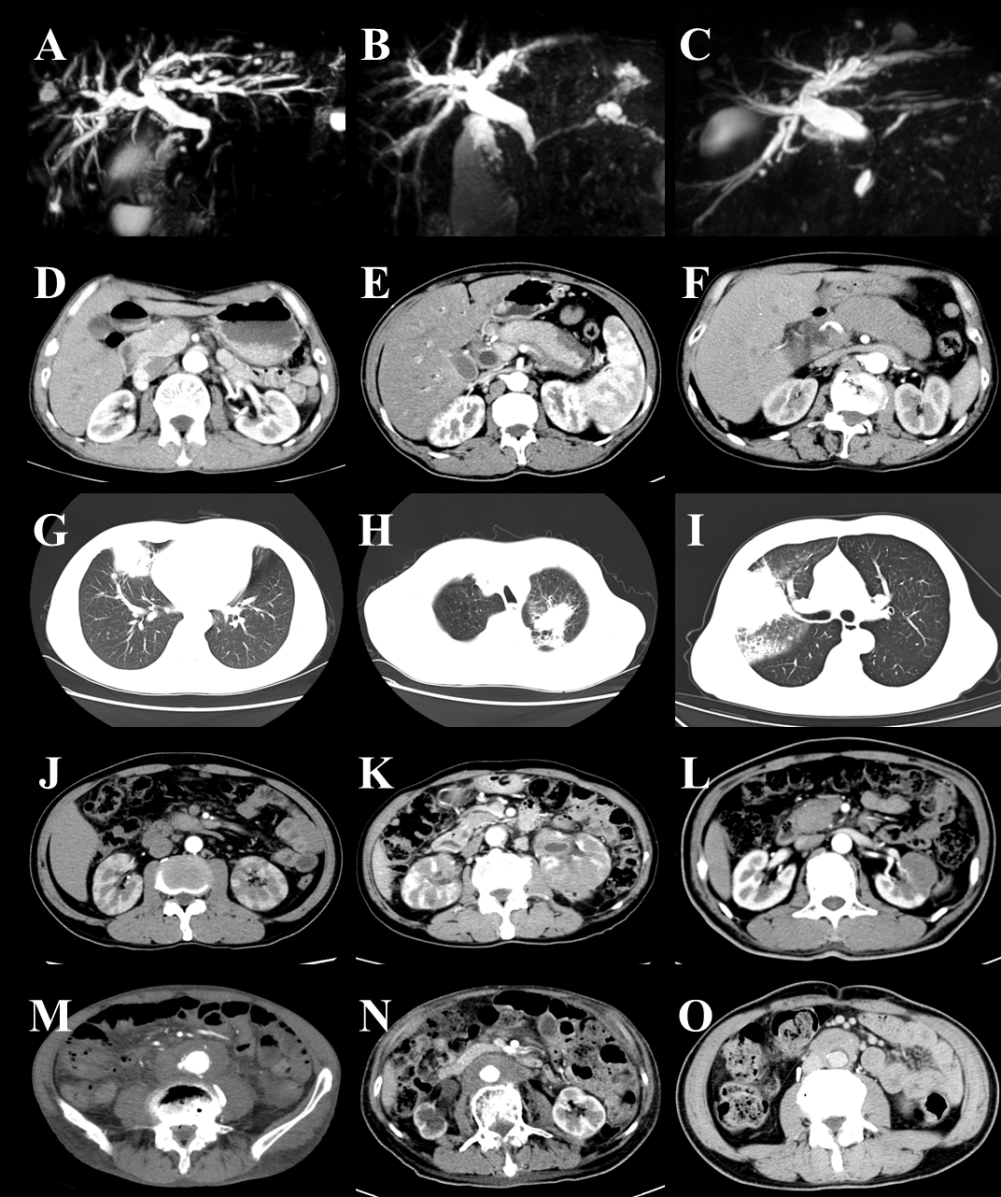
Features of Imaging in IgG4-RD. Bile duct: narrowing of the distal common bile duct **(A–C)**. Pancreas: focal enlargement of pancreatic head **(D)**, diffuse pancreatic enlargement **(E–F)**. Lung: solid nodule **(G)**, patchy infiltrate **(H–I)**. Kidney: hypodense shadow **(J–L)**.Retroperitoneal fibrosis: diffuse retroperitoneal involvement **(M–N)**, encasement of the aorta **(O)**

 All 40 patients with IgG4-related autoimmune pancreatitis underwent imaging studies, including CT, MRI, or MRCP scans (Figure 2A-F). Twenty-three patients (57.50%) exhibited diffuse pancreatic enlargement, with the majority displaying a ‘sausage-like’ appearance. Seventeen patients (42.50%) exhibited focal pancreatic enlargement, with 15 patients (88.24%) affected in the pancreatic head, one patient (5.88%) with involvement of the uncinate process, and one patient (5.88%) with involvement of both the pancreatic head and the uncinate process. Additionally, 11 patients (27.50%) presented with pancreatic duct dilation, and one patient (2.50%) had a pancreatic pseudocyst. Furthermore, 20 patients (50.00%) with IgG4-related autoimmune pancreatitis were concurrently diagnosed with IgG4-related sclerosing cholangitis (IgG4-SC). Among these, 7 patients (35.00%) exhibited involvement of the distal common bile ducts, and 5 patients (25.00%) had both distal common bile ducts and proximal intrahepatic bile ducts involvement, accompanied by dilation of the proximal common bile ducts. Additionally, 6 patients (30.00%) showed involvement of the distal common bile ducts and extensive intrahepatic bile ducts, also with dilation of the proximal common bile ducts, while 2 patients (10.00%) had exclusive involvement of the hilar bile ducts.

 In addition, there was one case of an IgG4-RD patient with isolated biliary involvement, as confirmed by imaging studies indicating exclusive involvement of the hilar bile ducts.

####  Lung 

 All 17 patients with IgG4-related lung disease underwent CT scans (Figure 2G-I). Among these, 8 patients (47.06%) exhibited solid nodules, 5 patients (29.41%) presented with patchy infiltrates, one patient (5.88%) demonstrated interstitial changes in the alveoli, one patient (5.88%) showed both broncho-vascular and patchy infiltrates, one patient (5.88%) revealed a combination of patchy infiltrates and interstitial changes in the alveoli, and one patient (5.88%) exhibited both solid nodules and interstitial changes in the alveoli.

####  Kidneys and Retroperitoneal Fibrosis

 Among 9 patients diagnosed with IgG4-related kidney disease, 5 patients underwent CT scans (Figure 2J-L). The imaging findings for all patients (100%) revealed hypodense shadows in the kidneys.

 All 6 patients with IgG4-related retroperitoneal fibrosis underwent CT scans (Figure 2M-O). Imaging findings revealed that 2 patients (33.33%) presented with encasement of the aorta, while 4 patients (66.67%) demonstrated diffuse retroperitoneal involvement.

## Discussion

 Despite the increasing recognition of IgG4-RD within the medical community, there remains a significant gap in the literature regarding a comprehensive comparison of the characteristics associated with multi-organ involvement versus single-organ involvement. Therefore, a systematic and comprehensive analysis of the clinical characteristics associated with multi-organ versus single-organ involvement in IgG4-RD will enhance clinicians’ understanding of the differences between these two patient groups. This study provides a critical foundation for the development of personalized treatment strategies, ultimately improving overall patient prognosis.

 In this study, we observed that 35 patients (42.68%) exhibited single-organ involvement, whereas 47 patients (57.32%) demonstrated multi-organ involvement. The most commonly involved organs were the lymph nodes, pancreas, and bile duct. These observations align with findings reported in previous studies.^24-27^ Our study also demonstrated that male patients exhibited a higher predisposition to multi-organ involvement in IgG4-RD. Previous studies have also confirmed this finding.^27-29^ This highlights the necessity for clinicians to adopt more individualized treatment strategies. Male patients, in particular, may require closer monitoring and early intervention to mitigate potential complications. Meanwhile, we found that patients with multi-organ involvement were more likely to be misdiagnosed with other conditions, highlighting a persistent gap in clinicians’ awareness of IgG4-RD. The diagnosis of IgG4-RD with multi-organ involvement is relatively complex and can be easily confused with other diseases. Therefore, clinicians should maintain a heightened level of vigilance during diagnosis to prevent misdiagnosis.

 In our study, we analyzed the incidence of additional organ involvement among patients with various types of involved organs and discovered that patients with involvement of the pancreas or lymph nodes exhibited a higher likelihood of concurrent involvement in additional organs. Meanwhile,it was particularly noteworthy that patients with IgG4-SC were frequently diagnosed concurrently with IgG4-related autoimmune pancreatitis, a finding that corroborated the conclusions of prior research.^30,31^ Therefore, clinicians should exercise heightened vigilance when evaluating patients with pancreatic or lymph node involvement. Regular multi-organ assessments, including imaging studies and laboratory tests, are essential for early identification of involvement in other organs. Such proactive monitoring aids in timely recognition of potential complications, ultimately enhancing patient prognosis. Public health agencies can also develop targeted screening guidelines based on the study findings, particularly for patients with pancreatic or lymph node involvement. Comprehensive health assessments should be recommended to facilitate early detection of multi-organ involvement in IgG4-RD. Certainly, the aforementioned findings require further researches for validation in order to elucidate the interactions among the multiple involved organs in IgG4-RD.

 We also conducted a statistical analysis of the laboratory findings between the multi-organ involvement group and the single-organ involvement group. In rheumatic and autoimmune diseases, eosinophils, ESR, and IgG function as pivotal biomarkers, playing crucial roles in inflammation, disease activity assessment, and immune modulation.^32-35^ In this study, we observed a significant elevation in the percentage and absolute count of peripheral blood eosinophils, serum IgG levels, and ESR in patients with multi-organ involvement compared to those with single-organ involvement. Additionally, our study demonstrated a moderate significant positive correlation between the number of involved organs in patients with IgG4-RD and ESR. We hypothesize that this phenomenon may be associated with the more pronounced systemic immune activation and inflammatory response resulting from multi-organ involvement. However, the correlations with eosinophil percentage, absolute eosinophil count, and serum IgG levels are relatively weak. We posit that this phenomenon could be attributable to the limited sample size. Overall, we hypothesize that these parameters may serve as early warning indicators of disease activity and severity, enabling physicians to promptly assess changes in the patient’s condition. In the future, we will further investigate the relationship between these parameters and the activity of IgG4-RD to clarify their roles in the pathogenesis and progression of the condition.

 Serum IgG4 levels are the most significant biomarker for IgG4-RD and serve as a critical parameter for disease diagnosis and evaluation.^22,23^ In this study, we categorized serum IgG4 levels into four groups (IgG4 ≤ ULN, ULN < IgG4 < 2 × ULN, 2 × ULN ≤ IgG4 < 5 × ULN and IgG4 ≥ 5 × ULN). Several studies have demonstrated a correlation between elevated serum IgG4 levels and an increased number of involved organs.^36,37^ In our study, we observed a weak significant positive correlation between the number of involved organs in patients with IgG4-RD and serum IgG4 levels. However, this study did not reveal a significant statistical difference in serum IgG4 levels between the multi-organ involvement group and the single-organ involvement group. This observation may be attributable to the fact that serum IgG4 levels were assessed as a semi-quantitative parameter and the sample size in this study was relatively limited. Although serum IgG4 levels did not exhibit significant differences between patients with multi-organ involvement and those with single-organ involvement, the weak correlation with the number of involved organs suggests that serum IgG4 levels may interact with other biomarkers to play a role in disease progression or the extent of organ involvement. Therefore, future studies should focus on elucidating the interplay between serum IgG4 levels and other biomarkers to achieve a more comprehensive understanding of its role in IgG4-RD.

 The current study has several limitations that warrant consideration. First, as a single-center retrospective analysis, the relatively limited sample size may potentially introduce selection bias. This bias arises from the possibility that the sample is not representative, particularly when analyzing subgroups. For instance, patients with specific characteristics or backgrounds may be underrepresented or overrepresented in our sample, which could compromise the accuracy and reliability of the results. Therefore, we should consider conducting multicenter studies in the future to increase the sample size and enhance the generalizability of the findings. Multicenter studies can encompass a broader patient population, reducing bias associated with single-center characteristics and improving the external validity of the results. Moreover, this study did not comprehensively evaluate the treatment strategies and prognostic outcomes for patients with multi-organ versus single-organ involvement in IgG4-RD. This gap may lead to an insufficient understanding of the clinical management of IgG4-RD patients. Future research should focus on comparing the treatment choices, and prognostic outcomes of these two groups to develop more personalized treatment strategies.

 In conclusion, IgG4-RD is characterized by its frequent involvement of multiple organs. Male patients exhibited a higher predisposition to multi-organ involvement in IgG4-RD. Our study indicated that patients with involvement of the pancreas or lymph nodes exhibited a higher likelihood of concurrent involvement in additional organs. Moreover, patients with multi-organ involvement exhibited significantly elevated levels of eosinophils, IgG, and ESR in peripheral blood. We also observed a moderate significant positive correlation between the number of involved organs and the ESR, while correlations with eosinophil percentage, absolute eosinophil count, serum IgG4 levels, and serum IgG levels were relatively weak. Therefore, the clinical features associated with multi-organ involvement in IgG4-RD are characterized by significant diversity and complexity. Clinicians must enhance their understanding of the characteristics associated with multi-organ involvement to more effectively improve patient prognosis.
